# Multifaceted Physiological Roles of Adiponectin in Inflammation and Diseases

**DOI:** 10.3390/ijms21041219

**Published:** 2020-02-12

**Authors:** Hyung Muk Choi, Hari Madhuri Doss, Kyoung Soo Kim

**Affiliations:** 1 Department of Clinical Pharmacology and Therapeutics, Kyung Hee University School of Medicine, Seoul 02447, Korea; chl2813@khu.ac.kr (H.M.C.); madhuridoss.h@gmail.com (H.M.D.); 2 East-West Bone & Joint Disease Research Institute, Kyung Hee University Hospital at Gangdong, Gandong-gu, Seoul 02447, Korea

**Keywords:** adiponectin, adiponectin isoform, pro-inflammatory, anti-inflammatory, rheumatoid arthritis, chronic kidney disease (CKD), inflammatory bowel disease (IBD)

## Abstract

Adiponectin is the richest adipokine in human plasma, and it is mainly secreted from white adipose tissue. Adiponectin circulates in blood as high-molecular, middle-molecular, and low-molecular weight isoforms. Numerous studies have demonstrated its insulin-sensitizing, anti-atherogenic, and anti-inflammatory effects. Additionally, decreased serum levels of adiponectin is associated with chronic inflammation of metabolic disorders including Type 2 diabetes, obesity, and atherosclerosis. However, recent studies showed that adiponectin could have pro-inflammatory roles in patients with autoimmune diseases. In particular, its high serum level was positively associated with inflammation severity and pathological progression in rheumatoid arthritis, chronic kidney disease, and inflammatory bowel disease. Thus, adiponectin seems to have both pro-inflammatory and anti-inflammatory effects. This indirectly indicates that adiponectin has different physiological roles according to an isoform and effector tissue. Knowledge on the specific functions of isoforms would help develop potential anti-inflammatory therapeutics to target specific adiponectin isoforms against metabolic disorders and autoimmune diseases. This review summarizes the current roles of adiponectin in metabolic disorders and autoimmune diseases.

## 1. Introduction

Adiponectin is a secretory protein produced by adipocytes of white adipose tissue [1]. Structurally, adiponectin consists of a nitrogen terminal collagen domain and a carboxyl terminal globular domain [2]. It belongs to the soluble collagen superfamily and is homologous to complement factor C1q and the tumor necrosis family (TNF) [3]. The biological functions of adiponectin are varied since it circulates in blood in multiple isoforms. It has three different molecular weights: low-molecular weight (LMW) (trimer), middle-molecular weight (MMW) (hexamer), and high-molecular weight (HMW) (multimer) [4]. Studies have reported that HMW adiponectin is involved in insulin sensitivity, glucose uptake, and lipid metabolism. Investigative research has reported the involvement of different adiponectin isoforms in the pathogenesis of various diseases [5]. It has also been shown that these isoforms act as acute-phase reactants that influence inflammatory mechanisms in both acute and chronic diseases. Disruption in the formation of adiponectin isoforms is one of the major dispositions for metabolic disorders [6]. Research over the years has revealed the bilateral functions of adiponectin, which have since become a matter of conflict among different researchers. The existing duality in adiponectin (i.e., having both pro-inflammatory and anti-inflammatory effects) is known to contribute to the pathogenesis of a number of diseases [7,8,9,10]. A lack of understanding of these phenomena might be attributed to the inability to accurately measure adiponectin isoforms or the absence of a universal standard. In this review, we provide a detailed overview of the physiological roles of adiponectin and its related isoforms. We highlight their role in inflammation and other related diseases with a focus on its dual behavior in different pathological conditions.

## 2. Adiponectin

### 2.1. Brief Overview of Adipose Tissue and Adiponectin Biology

Adiponectin is the most abundant adipokine in human blood with a physiological level of 5–30 μL/mL secreted from adipose tissues [1]. There are two kinds of adipose tissue present in humans: white adipose tissue (WAT) and brown adipose tissue (BAT). Adiponectin (also known as Acrp30, GBP-28, apM1, and AdipoQ) is a protein mainly secreted by WAT adipocytes. Other tissues such as human murine osteoblasts, liver, parenchyma cells, myocytes, epithelial cells, and placental tissue show low levels of adiponectin secretion [2]. The main biological functions of adiponectin include enhanced fatty acid biosynthesis and inhibition of gluconeogenesis in the liver [3]. In addition, it enhances glucose uptake in skeletal muscle via signaling pathways. Studies have shown that adiponectin can be used to improve insulin resistance by reducing the amount of intracellular fat through increased oxidation of fatty acid via PPARα activation and enhancement of insulin receptor substrate (IRS) signaling in skeletal muscles and the liver [4,5]. Furthermore, adiponectin has been reported to possess antioxidant, anti-inflammatory, and anti-atherosclerotic effects [6].

### 2.2. Adiponectin Isoform

**Figure 1:**
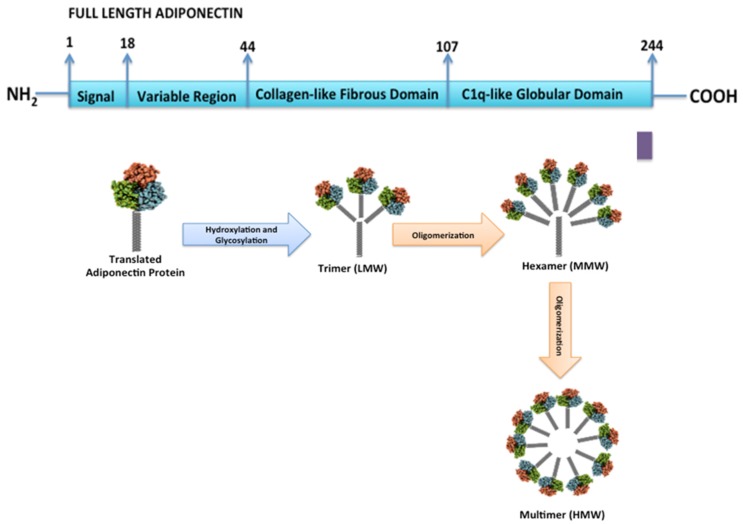
Domains and structure of adiponectin. The primary structure of adiponectin, which consist of 244 amino acids, has an N-terminal signal sequence, a variable region with attached O-glycoside side chains, a collagenous domain, and a C-terminal sequence of a globular domain.

Human adiponectin is encoded by the Adipo Q gene, which spans 15.8 kb on chromosome locus 3q27. The human adiponectin gene contains three exons with the start codon in exon 2 and stop codon in exon 3 [7]. As shown in Figure 1, full length human adiponectin, has 244 amino acids, and consists of four regions including signal sequence at the N-terminus of 18 amino acids, a variable region of 24 amino acids, a collagenous domain of 65 amino acids, and a C-terminal globular domain of 137 amino acids [8,11]. After synthesis, it undergoes post-translational modification by glycosylation and hydroxylation.

During this process, adiponectin creates three major oligomeric isoforms: LMW trimer, MMW hexamer, and HMW multimer [9]. Several conserved lysine residues (Lys65, Lys68, Lys77, and Lys101 for human adiponectin) within the N-terminal collagenous domain of adiponectin are modified by hydroxylation and subsequent glycosylation. In addition, proline residues (Pro71, Pro76, and Pro95 for human adiponectin) are hydroxylated, and a cysteine residue at the N-terminus (Cys36 for human adiponectin) forms an intermolecular disulfide bond. Post-translated adiponectin forms three functional isoforms.

These isoforms are formed through a series of complex signaling and modification processes. The LMW (60 kDa) is a trimer of three adiponectin monomers (28 kDa) formed by joining the C-terminal globular domain and the collagen-like domain. The trimers further multimerize to form MMW (150 kDa) hexamers and HMW (420 kDa) multimers, which are comprised of 12–32 monomers. Among the adiponectin isomers, MMW and HMW constitute the majority of circulating adiponectin. LMW monomers are usually not detected in circulation but are present at very low concentrations in human plasma [12].

### 2.3. Different Physiological Roles of the Adiponectin Binding Receptors of AdipoR1, AdipoR2, and T-Cadherin

Adiponectin receptors AdipoR1 and AdipoR2 are composed of an intracellular NH_2_-terminal domain and an extracellular COOH-terminal domain. These receptors have seven transmembrane domains that are distinct from the G-protein coupled receptors. AdipoR1 and AdipoR2 are significantly homologous, and show 67% amino acid identity, but have different affinities for full-length adiponectin and globular adiponectin [10]. For example, small-interfering RNA transfection (siRNA) induced suppression of *AdipoR1* and *AdipoR2* showed that AdipoR1 had high-affinity for globular adiponectin, and AdipoR2 had intermediate affinity for both full-length adiponectin and globular adiponectin. In addition, the tissue expression profile of *AdipoR1* and *R2* showed AdipoR1 was ubiquitously expressed in skeletal muscle and the liver, while AdipoR2 was mainly expressed in the liver [13]. Transgenic mice overexpressing AdipoR1 and AdipoR2 showed enhanced ceramidase activity, glucose metabolism, and insulin sensitivity. This suggests that the receptors are important in regulating metabolic activities [14]. More importantly, knock-out of *AdipoR1* and *AdipoR2* genes resulted in increased fat deposits in tissues, inflammation, oxidative stress, and insulin resistance. Other studies showed that disruption of adiponectin receptors affected the normal functioning of signaling molecules. *AdipoR1* disruption decreased adiponectin-induced AMPK activation, while disruption of *AdipoR2* resulted in decreased PPARα activation. These outcomes are a clear indication that AdipoR1 is associated with activation of AMPK signaling pathways [15].

T-cadherin works as a receptor for HMW and MMW but not for globular or LMW adiponectin in specific tissues like muscle [16]. Numerous studies support the association between adiponectin and T-cadherin. The siRNA transfection-induced *T-cadherin*-deficient mice did not show adiponectin-mediated revascularization. *T-cadherin* deficiency disrupted adiponectin’s ability to promote cellular migration and proliferation. Furthermore, T-cadherin seems to be specifically involved in adiponectin-stimulated muscle regeneration. The regeneration of myofibers increased in adiponectin-overexpressing mice but not in null *T-cadherin* mice [12]. Consistent with these results, adiponectin-mediated exosome production was less effective in differentiating C2C12 in vitro when *T-cadherin* was knocked down [17]. All these results show a possible link between T cadherin and adiponectin receptors that need to be further explored.

## 3. Signaling Proteins in Adiponectin-Stimulated Signaling Pathways

### 3.1. Adaptor Protein Containing a Pleckstrin Homology Domain (APPL) 1 Protein

**Figure 2:**
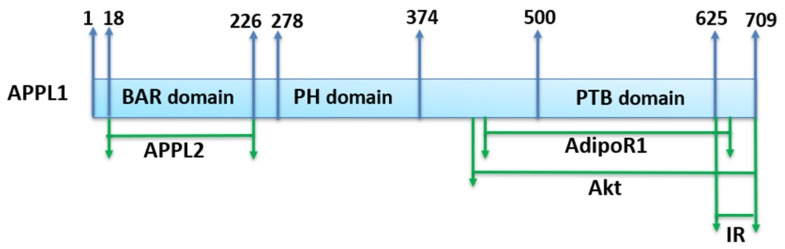
A broad APPL1 domain structure and binding sites of interaction proteins. The primary structure of APPL1, which consist of 709 amino acids, has a BAR (Bin1/Amphiphysin/RVS167) domain, a PH (pleckstrin homology) domain, and a PTB (phosphotyrosine binding) domain. Broad sites of interacting proteins with APPL1 are labeled in green.

APPL1, is the first identified protein that interacts directly with adiponectin receptors [18]. This type of interaction plays a key role in molecular signaling as an adaptor protein that binds to AdipoR1, AdipoR2, insulin receptors, and other signaling proteins (Figure 2). When AdipoR1 is stimulated by adiponectin as the COOH-terminal domain of AdipoR1 interacts with adiponectin, intracellular NH_2_-terminal domain of AdipoR1 interacts with the phosphotyrosine binding (PTB) domain of APPL [18]. It is well established that APPL1 mediates several signaling cascades including serine/threonine kinase Akt, phosphatidylinositol-4,5-biophosphate 3-kinase (PI3K), insulin receptor substrate proteins 1, 2 (IRS1/2), adenosine monophosphate-activated protein kinase (AMPK), and mitogen-activated protein kinase (MAPK) by a direct interaction with membrane receptors [19,20,21,22,23].

The role of APPL1 in IRS1/2-IR (insulin receptor tyrosine kinase) activation seems to be related to insulin sensitivity [22]. IRS1/2 must form a complex with APPL1 in order to bind to the cytoplasmic domain of the insulin receptor (IR), which can regulate insulin sensitivity through the signaling pathway of IR. In contrast, systemic insulin resistance was observed in cases of APPL1-/- mice, and insulin stimulation did not increase the expression level of IRS1/2. In-silico analyses have reported that activated IRS1/2 provides docking sites to p85 PI3K for the activation of PI3K [23]. APPL1 phosphorylates AKT2, which is a key molecule in the insulin signaling pathway, which prompts insulin-induced glucose metabolism. Familial diabetes patients with mutations on APPL1 showed a problem in AKT2 phosphorylation and insulin signaling [24]. Mice with knock-down of IRS1/2 or IR also showed remarkable downregulation of insulin-stimulated AKT phosphorylation and decreased glucose uptake. Furthermore, the mice demonstrated downregulated secretion of adiponectin, which activates PI3K-Akt signaling [25]. This means that insulin resistance could increase in mice due to scarcity of adiponectin because of the involvement of PI3K-Akt signaling insulin resistance. Another theory also indicates IRS1/2-PI3K-Akt signaling, which is activated by an interaction between adiponectin and APPL1 lowering insulin resistance [26]. A recent review suggested a disturbed insulin sensitizing action of adiponectin along with APPL1 as a major factor in type 2 diabetes [27].

The role of adiponectin is implicated in a number of essential regulatory cellular signaling mechanisms including AMPK, p38 MAPK signaling, and lipid metabolic pathways. Adiponectin initiates APPL1-AMPK signaling that translocates transcription factors into the nucleus. The siRNA-mediated knockdown of *APPL1* showed down-regulated phosphorylation of AMPK upon stimulation with adiponectin [28]. In addition, a recent study indicated that highly conserved 13-residue segment from an adiponectin collagen domain induced interaction between APPL1 and Rab5. Interaction between APPL1 and Rab5 induces the phosphorylation of AMPK, MAPK, and activation of peroxisome proliferator-activated receptor (PPAR) α. Phosphorylation of the above key signaling proteins showed enhanced adipocyte differentiation, glucose uptake, and lipid metabolism [20]. Another theory also suggests an adiponectin-APPL1 interaction suppresses glucogenesis. The involvement of APPL1, in mediating downstream effects of adiponectin through adiponectin receptors in the liver, is still unclear. However, in mouse hepatocyte cells overexpressing *APPL1* showed p38 MAPK activation, which suggests a role of APPL1 in adiponectin signaling [18]. Similar studies have demonstrated protein levels of APPL1 were negatively related with mRNA levels of gluconeogenesis enzymes. In addition, adiponectin induced phosphorylation of STAT3 was positively influenced by APPL1 [29]. In addition, APPL1 regulated adiponectin-induced fatty acid oxidation in cardiomyocytes. Adiponectin increased the binding of APPL1 with AMPK in vitro and also increased the oxygen consumption of cardiomyocytes [30]. Adiponectin mediated APPL1-AMPK signaling also attenuated neuronal apoptosis in hypoxia-ischemia induced rats. In the study, recombinant human adiponectin administration to hypoxia-ischemia induced rats revealed a decrease in the brain atrophy area, neuronal apoptosis, and simultaneously increased AdipoR1, APPL1, and p-AMPK expression compared to controls [31]. Additionally, research studies have shown that adiponectin signaling through APPL1 is required to exert its anti-inflammatory effects in endothelial cells [32]. Similarly, hematopoietic cell from *APPL1*-specific deficient mice showed increased ROS generation, inflammasome activation, and insulin resistance. Likewise, in vitro experiments involving interrupted APPL1 activation in macrophage showed high level of ROS and IL-1β secretion [27]. The p38 MAPK pathway is also stimulated by APPL1, which is activated by adiponectin. This pathway is involved in cell cycle regulation, inflammation, and apoptosis [33]. APPL1 also activates ligand-dependent transcriptional regulator *PPAR-α*, which regulates the expression of genes that are vital to lipid metabolism. It is also involved in clearance of cellular/circulating lipids in liver and skeletal muscles [34,35].

### 3.2. Adaptor Protein Containing Pleckstrin Homology Domain (APPL) 2 Protein

APPL2 is an isoform of APPL1, and they share 54% identical protein sequences. APPL1 and APPL2 are structurally similar and contain an N-terminal BAR domain, a central PH domain, and a C-terminal PT domain. APPL2 is essential for cell proliferation and embryonic development. A recent study indicated that APPL2 forms dimers with APPL1 via the BAR domain. APPL2 initiates follicle stimulation hormone signaling by forming a complex with the FSH receptor, APPL1, and Akt 2 [36]. Another study showed that APPL2 functions as a negative regulator in adiponectin signaling by competitively binding to the intracellular domain of AdipoR1 against APPL1 and, thereby, blocking adiponectin signaling in muscle cells [37]. Overexpression of *APPL2* synergistically blocks adiponectin on insulin-stimulated Akt activation, which indicates that APPL isoforms function as dual regulators in adiponectin signaling.

### 3.3. Signaling Pathways of Adiponectin-Mediated Metabolism

**Figure 3:**
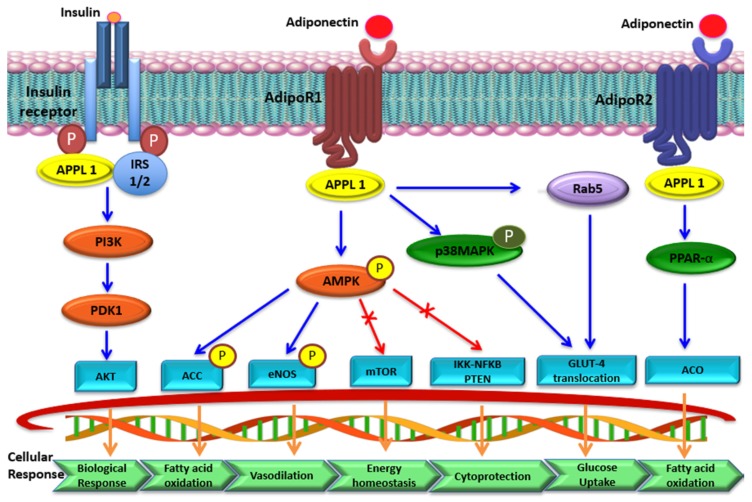
Key signaling pathways of adiponectin. Adiponectin and AdipoRs (AdipoR1 and AdipoR2) interact to activate downstream signaling pathways. Binding of adiponectin to its receptors activates adaptor protein APPL1. Activated APPL1 initiates complex signal transduction by activating PPAR-α and phosphorylating AMPK and p38-MAPK. Phospho-AMPK inhibits lipogenesis and promotes fatty acid oxidation and transport into the mitochondria by phosphorylating ACC-1. Phosphorylated eNOS stimulates nitric oxide (NO), which results in vasodilation. In addition, adiponectin shows cytoprotective effect because activation of AMPK suppresses mTOR and IKK-NF*-*κB-PTEN signaling. Metabolic effects of insulin are mainly controlled by PI3K-Akt signaling. As PI3K-Akt is activated, glycogen synthesis and glucose uptake increases but lipolysis is suppressed. Insulin sensitivity increases when IRS1/2 is activated by adiponectin.

Adiponectin signaling mainly relies on receptor-ligand type interactions wherein adiponectin binds to its cognate receptors and initiates the activation of several intracellular signaling cascades through AMPK, mTOR, NF-κB, STAT3, and JNK pathways [38]. As shown in Figure 3, adiponectin initiates activation of AMPK signaling mediated by the APPL1 adapter protein, which binds to the intracellular domain of AdipoR. AMPK activation leads to activation of related downstream targets, which include biosynthesis of molecules, other regulatory proteins, and important transcription factors. AMPK is an upstream regulator of mTOR that is mainly involved in cell proliferation and cancers [39].

In vitro studies showed that adiponectin increases both AMPK and acetyl-CoA carboxylase (ACC) phosphorylation, which, in turn, activates key enzymes for β-oxidation in rabbit blastocysts [40]. Adiponectin is known to stimulate the expression of transcription of *fatty acid transporter protein 4 (FATP4*) and *fatty acid binding protein (FABP)* through AMPK signaling for activation of β-oxidation enzymes. In addition, adiponectin is known to modulate the function of endothelial progenitor cells via AMPK-eNOS activation, which plays a pivotal role in vascular protection by differentiating them to endothelial cells [41]. Lastly, the adiponectin-AMPK-eNOS signaling pathway plays an important role in promoting endothelial cell proliferation and cell migration. It was demonstrated that adiponectin might be useful in treatment of obesity-related vascular deficiency diseases through adiponectin-AMPK-eNOS signaling activation [42]. Additionally, adiponectin increases production of nitric oxide (NO) through dephosphorylation of Caveolin-1 and phosphorylation of eNOS. It inhibited abnormal proliferation of human aortic endothelial cells, which helps prevent cardiovascular diseases [43]. Adiponectin also helps to improve inflammatory cardiomyopathy by decreasing reactive oxygen stress (ROS) [44]. Based on a study of patients with inflammatory cardiomyopathy, patients with a high adiponectin level showed better ventricular protection and a low level of inflammation. In experimental autoimmune myocarditis mice, adiponectin suppressed TNF-α-mediated NF-κB activation and production of ROS.

## 4. Beneficial Effect of Adiponectin in Metabolic Tissues

**Figure 4:**
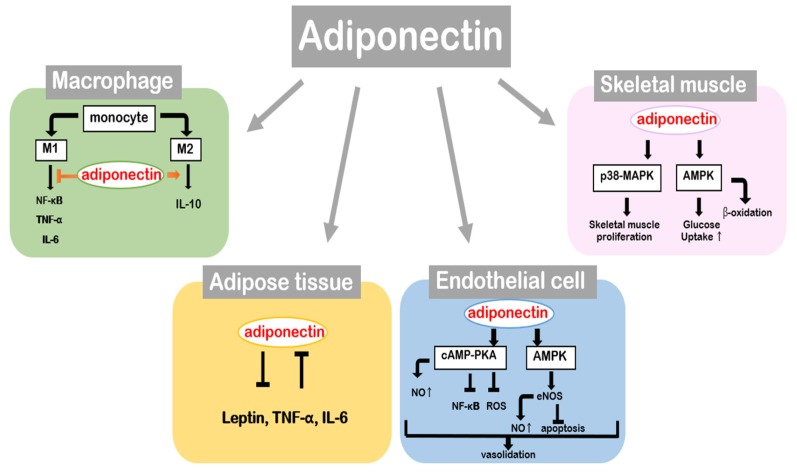
Physiological roles of adiponectin in the main tissues.

Adiponectin functions as an insulin sensitizer and exhibits anti-diabetic, anti-inflammatory, and anti-atherogenic effects. Its multifunctional aspects render it a highly favorable target for metabolic disorders [45]. The central role of adiponectin is energy homeostasis with a newly proposed role as a “starvation gene” [46]. The following section explains adiponectin-mediated cellular signaling in different tissues and their associated effects (Figure 4).

Adiponectin seems to mediate various tissue-specific signaling pathways. On macrophages, adiponectin promotes cellular differentiation of monocytes to M2 macrophages and suppresses their differentiation to the M1 macrophages, which shows proinflammatory and anti-inflammatory effects, respectively. Adipose tissue releases various adipokines, and adiponectin is mainly involved in controlling the endocrine system of adipose tissue. Adiponectin represses secretion of leptin and proinflammatory cytokines including IL-6 and TNF-α, which lower the expression level of adiponectin. On endothelial cells, adiponectin induces cAMP-PKA and AMPK to regulate vascular homeostasis. Enhanced COX-2 and eNOS activity and subsequent nitric oxide (NO) production improve endothelial cell function and block secretion of inflammatory factors. On skeletal muscle, adiponectin stimulates AMPK to enhance fatty acid oxidation and glucose uptake. In addition, crosstalk between insulin and adiponectin signaling showed that adiponectin synergistically improves insulin sensitivity and glucose tolerance. The p38-MAPK pathway of adiponectin is known to stimulate the proliferation of skeletal muscle tissue.

### 4.1. Effect of Adiponectin on Adipose Tissue

Adipose tissue plays an integral role in regulating energy metabolism and glucose homeostasis. These functions are active at both organ and systemic levels [47]. The adipose tissue is comprised of a variety of cell populations including macrophages, endothelial cells, fibroblasts, and leucocytes. Adipose tissue exerts its endocrine effects by releasing adipokines that act as chemical messengers, which communicate with other organs and control a range of metabolic signals. In addition, adipose tissue plays an indispensable role in lipid mobilization and energy distribution in the body [48]. Treatment with pro-inflammatory cytokines like IL-6 and TNF-α is known to decrease secretion of adiponectin in 3T3L1 adipocytes. Consistent with in vitro results, obese people who have high levels of IL-6 and TNF-α showed decreased secretion of IL-6 and TNF-α with an increased adiponectin level [49]. In reference to the above AMPK pathway, adiponectin first activates AMPK signaling, and then suppresses the IKK-NF-κB-PTEN signaling pathway. Lastly, adiponectin aids in suppressing *IL-6* and *TNF-α* expression, which is activated by NF-κB [50]. Furthermore, the adiponectin level is known to have a negative correlation with both IL-6 and a high-sensitivity C-reactive protein [51]. This can also be explained by the mechanism discussed above.

### 4.2. Effect of Adiponectin on Skeletal Muscle

Studies have demonstrated a critical role for adiponectin in skeletal muscle tissues. Secretion of adiponectin in skeletal muscle fluctuates with exercise and diet. For example, exercise decreases the level of full-length adiponectin, which leads to an increase in muscle function through decreased inflammation and insulin resistance [52]. Adiponectin also seems to have anti-inflammatory and insulin-sensitive effects on skeletal muscle tissue. Adiponectin knockout mice showed decreased miR-711 expression, while mice overexpressing adiponectin showed elevated miR-711 expression in muscle tissue. MiR-711 overexpression leads to repression of NF-κB through the toll-like receptor (TLR)-4 pathway. This study indicated that mir-711 has anti-inflammatory effects that might be induced by adiponectin in muscle tissue [35]. However, a high level of adiponectin is not always good for skeletal muscle health. Phosphorylation of AMPK is triggered by signaling of AdipoR1 and AdipoR2. AMPK is also phosphorylated by AdipoRon, which is a synthetic, small molecule agonist for AdipoR1 and AdipoR2. At a high dosage, AdipoRon unfavorably suppresses the protein content, myotube diameter, and number of nuclei per myotube of C2C12 cells. This indirectly suggests that an abnormally high level of adiponectin may have detrimental effects on muscle cells by excessively activating AdipoR signaling [53].

### 4.3. Effect of Adiponectin on Vascular Endothelium

AMPK-endothelial nitric oxide synthase (eNOS) activation promotes NO production, which contributes to vascular protection and endothelium relaxation [54]. In addition, adiponectin activates protein kinase A (PKA) signaling, which promotes NO production and suppresses ROS generation and NF-κB signaling. Adiponectin knockout mice showed a decreased mRNA level of *NOS* and a production level of PGE_2_. Treatment with adiponectin reversed the decreased levels of NOS and PGE_2_ in *adiponectin*-KO mice and also lowered blood pressure [55]. eNOS is a key signaling molecule involved in the vasculo-protective role of adiponectin. Increased NO production through eNOS activation reduces the risk of vasoconstriction, as NO blocks plaque formation and endothelial apoptosis [56]. Especially in cardiovascular cells, adiponectin activates AMPK and COX-2 pathways, which blocks secretion of LPS-induced TNF-α. This stimulates atherosclerotic proliferation. As a result, adiponectin showed anti-inflammatory and anti-apoptotic effects on cardiac cells [40]. Adiponectin-induced AMPK-COX-2-PGE_2_ suppressed mRNA expression of *IL-8* and *vascular cell adhesion molecule-1* [41].

Adiponectin-induced COX-2 activation seems to stimulate endothelial cell migration. When endothelial cells from hind limbs of mice were treated with adiponectin, endothelial cell migration increased with *COX-2* expression. However, *COX-2* deficient mice did not show a similar effect after treatment with adiponectin [42]. This implies that adiponectin-induced AMPK signaling can restore impaired angiogenesis, which is a risk factor for cardiovascular disease. However, adenovirus-mediated dominant-negative AMPK injection decreased the degree of perfusion enhancement by adiponectin [57]. In contrast, another study indicated that adiponectin-induced angiogenesis is promoted by crosstalk of AMPK and Akt signaling. Specifically, transduction of dominant-negative AMPK or dominant-negative Akt, blocked both eNOS phosphorylation and adiponectin-stimulated human umbilical vein endothelium cell migration [58]. Additionally, T-cadherin, which is considered as a potential adiponectin receptor, is essential for adiponectin-induced revascularization. Cultured endothelial cells whose *T-cadherin* was knocked down by siRNA showed diminished adiponectin-induced cellular migration and proliferation [12].

### 4.4. Effect of Adiponectin on Macrophages

There are two types of macrophages: M1 and M2. M1 is known to stimulate pro-inflammatory factors and induce insulin resistance. In contrast, M2 is known to block an inflammatory response and promote oxidative metabolism. Adiponectin is known to interrupt M1 macrophage activation and support M2 macrophage activation [59]. Adiponectin also activates anti-inflammatory factors IL-10 but reduces proinflammatory cytokines such as IFN-γ, IL-6, and TNF-α in human macrophages [60]. Adiponectin plays an anti-inflammatory role by regulating JmJC family histone demethylase 3 (JMJD3), which is instrumental for M2 polarization. In experiments on diet-induced obese mice whose *JMJD3* expression is decreased, treatment with adiponectin showed up-regulated *JMJD3* expression and reduced macrophage infiltration in adipose tissue. Consequently, adiponectin contributed to anti-inflammatory macrophage polarization and blocked macrophage infiltration [61]. The above results indicate adiponectin’s anti-inflammatory effects by suppressing M1 and stimulating M2.

However, some studies showed opposing results. HMW and globular adiponectin activated TNF-α secretion in human monocytic cells. Mutated adiponectin that cannot form HMW could not stimulate TNF-α. Additionally, when treatments were conducted with globular adiponectin, mRNA expression of inflammatory marker genes significantly increased [62]. However, globular adiponectin is known to induce *TNF-α* and *IL-6* in macrophages. Another study showed that it produces macrophage resistance to inflammatory stimuli [63]. This indicated that globular adiponectin might have an instant pro-inflammatory effect, which can be quickly alleviated.

On the one hand, the M1 phenotype could inhibit expression of adiponectin receptors. For example, the M1 macrophage suppressed 40–60% of AdipoRs expression compared to controls, whereas M2 macrophage preserved it [64]. However, adding adiponectin to M1 macrophage induces the production of anti-inflammatory cytokines IL-10 rather than pro-inflammatory cytokines, while M1 macrophage normally expresses pro-inflammatory cytokines such as TNF-α, IL-6, and IL-12. These results indirectly show that adiponectin can regulate macrophage activation and production of pro-inflammatory cytokines.

### 4.5. Insulin Sensitizing Action of Adiponectin

Insulin sensitizing activity of adiponectin is mediated through interactions with its AdipoR1 receptor via activation of the AMPK pathway [65]. Three independent groups first identified this effect of adiponectin [66,67,68]. It was reported that replenishment of adiponectin showed a significant improvement in diet-induced insulin resistance and hypertriglyceridemia in high-fat diet-fed KKAv mice. This led to the hypothesis that adiponectin serves as an insulin sensitizing adipokine. High-fat diet-induced hypoadiponectinemia has been strongly associated with insulin resistance and metabolic syndromes [66]. In vivo studies have determined that an increase in the adiponectin level triggers a transient decrease in glucose by inhibiting expression of gluconeogenic enzymes in both wild-type and type 2 diabetic mice [67]. Studies have reported the presence of very small amounts of a truncated/proteolytically cleaved form of adiponectin (consisting of the globular domain) in plasma [68]. Lodish and colleagues reported that the truncated product of adiponectin is involved in increased fatty acid oxidation in muscle tissues and reduction in plasma glucose and considerable weight loss in experimental mice [68]. Further investigations to assess the effects of adiponectin on insulin resistance were carried out in *adiponectin* transgenic mice [69] and *adiponectin*-deficient mice [70,71,72]. However, some studies have shown contradicting effects of adiponectin in deficient mice. The *adiponectin* deficient mice showed a mild insulin resistance with glucose intolerance while on a standard diet [73]. Another study exhibited almost normal insulin resistance in normal diet-fed mice [71]. Furthermore, mice fed a high-fat, high-sucrose diet showed severe insulin resistance, especially in skeletal muscle [74]. It was reported that fatty acid oxidation substantially increased in skeletal muscle of *adiponectin*-deficient mice, but there was no effect on insulin sensitivity or glucose tolerance. All these studies suggest that adiponectin plays a significant role in regulating insulin sensitivity.

## 5. Is Adiponectin Anti-Inflammatory or Proinflammatory?

Circulating serum level of adiponectin is decreased in patients with type 2 diabetes, metabolic syndrome, or cardiovascular disease [38]. Extensive research has shown that adiponectin possesses anti-inflammatory properties [75]. This was the common view, but recent reports have offered contradictory findings that adiponectin also has pro-inflammatory aspects in some diseases [76]. The pro-inflammatory or anti-inflammatory role of adiponectin is reviewed in the context of various inflammatory diseases in this section.

### 5.1. Diseases Associated with Anti-Inflammatory Effects of Adiponectin

Adiponectin plays a critical role in energy metabolism. As mentioned previously, adiponectin activates glucose transport and inhibits gluconeogenesis through AMPK signaling and oxidizes fatty acid by the PPARα pathway. However, obesity decreases adiponectin secretion [77]. When people lose weight, the adiponectin level increases, and this has a positive relationship with BMI (body mass index) reduction based on experiments conducted on 22 obese people [78]. A recent study indicated that adipose tissue of obese people shows impaired leptin signaling and increased caveolin-1 expression, which reduces adiponectin secretion [79]. Additionally, leptin and adiponectin imbalance is associated with BMI and decreased vascular response. Leptin and adiponectin have opposing reactions. Leptin up-regulates pro-inflammatory factors like TNF-α and IL-6, while adiponectin down-regulates them [80]. Therefore, obesity or obesity-related diseases like type 2 diabetes are closely related to the adiponectin serum level. In addition, a decreased level of adiponectin brings about many physiological changes and is closely related to insulin resistance and cardiovascular and inflammation associated diseases [38].

#### 5.1.1. Anti-Inflammatory Role of Adiponectin in Atherosclerosis

Atherosclerosis is a chronic disease of the arterial walls and is associated with inflammation. A low level of adiponectin is known to increase the risk of atherosclerosis [81,82]. Adiponectin injection increased the gene expression of anti-inflammatory factors eNOS and IL-10. *Apolipoprotein E*-deficient mice (*ApoE-/-*) mice have adiponectin deficiency. In addition, exogenous adiponectin reduced gene expression of pro-inflammatory factors like *TNF-α*, *IL-6*, and *vascular cell adhesion molecule-1 (VCAM-1)*. Additionally, adiponectin effectively inhibited the activation of NF-κB pathway and expression of NF-κB nuclear protein p65 [83]. All these results indirectly demonstrate that adiponectin induces anti-inflammatory effects.

On the other hand, lipid accumulation induces low-grade chronic inflammation [84], and adiponectin seems to inhibit accumulation of lipids in an atherosclerosis animal model of high-fat *ApoE-/-*mice, which have adiponectin deficiency. Adenovirus-induced overexpression adiponectin mice showed decreased levels of cholesterol, triglyceride, and low-density lipoprotein cholesterol (LDL-C) [85]. Other experiments on *ApoE-/-*mice showed that adiponectin suppressed mRNA levels of *VCAM-1*, *class A scavenger receptor (SR-A), and TNF-α* [86]. In addition, mRNA expression of *eNOS* was significantly stimulated [85]. Recent experiments with *ApoE-/-*mice indicated that adiponectin blocked inducible nitric oxide synthase (iNOS). This means that adiponectin decreases oxidative stress [87]. In summary, adiponectin may control atherosclerosis by reducing inflammation, lipid accumulation, and oxidative stress.

#### 5.1.2. Anti-Inflammatory Role of Adiponectin in Obesity and Type 2 Diabetes

Obesity and type 2 diabetes patients show a decreased serum adiponectin level and low-grade chronic inflammation associated with an increased production of pro-inflammatory cytokines like IL-6 and TNF-α [88]. These pro-inflammatory cytokines interrupt normal signaling of insulin and β-cell function, which decreases symptoms of obesity and Type 2 diabetes [89]. Adiponectin repressed secretion of LPS-stimulated TNF-α in cultured human macrophages [90]. Globular adiponectin inhibited activation of TLR-mediated NF-κB [91], and adiponectin increased the secretion of IL-10 [92]. The increased IL-10 expression stimulated the production of matrix metalloproteinase-1 inhibitor in human macrophages, which alleviated tissue destruction [93]. The studies discussed above have identified a decreased adiponectin level to be associated with not only disturbed energy metabolism and vascular diseases, but also chronic inflammation through complicated signaling and interaction with other adipokines. Numerous studies revealed that n-3 polyunsaturated fatty acids (PUFAs) have an effect on leptin and adiponectin levels. Meta-analysis on 494 patients with type 2 diabetes from 10 articles concluded that plant and marine sources of n-3 PUFAs seem to boost adiponectin levels and decrease leptin [94]. Furthermore, n-3 PUFA supplementation in type 2 diabetes patients brought leptin levels down and increased the adiponectin level, which resulted in positive effects on lipid profile, insulin, and glycosylated hemoglobin [95]. Other double-blind randomized clinical trials of n-3 PUFAs also suggest that n-3 PUFAs reduced the serum MCP-1 level and atherogenic risk [96]. These studies imply that adiponectin has anti-inflammatory effects in the metabolic diseases described above.

### 5.2. Diseases Associated with Pro-Inflammatory Effects of Adiponectin

#### 5.2.1. Pro-Inflammatory Role of Adiponectin in Rheumatoid Arthritis (RA)

It is now well established that RA patients show high plasma levels of adipokines like adiponectin, leptin, and visfatin when compared to healthy controls [97]. A meta-analysis investigated 813 RA patients and 684 healthy controls in 11 studies and indicated that circulating adiponectin level of RA patients was significantly higher than that in control groups [98]. Comparing serum adiponectin isoform levels in RA patient groups with those in control groups, the HMW level in RA patients was higher than that of the control group, and the MMW levels were not different between the groups. In contrast, the globular adiponectin level in RA patients was much higher than that in the healthy control group [99]. Thus, it was suggested that HMW and globular adiponectin could be used as a potential biomarker for RA due to the considerable difference in the plasma adiponectin level in RA patients versus healthy individuals [100].

Despite the high serum level of adiponectin, extensive studies have shown that RA results in a systemic chronic inflammation [101]. Adiponectin stimulated production of pro-inflammatory factors such as IL-6, IL-8, and PGE_2_ in fibroblast-like synoviocytes (FLS) of RA patients [102]. In addition, adiponectin enhanced the production of VEGF (vascular endothelial growth factor) and MMPs (matrix metallopeptidases) in FLS of RA, which may lead to inflammation and joint destruction [103,104]. Adiponectin dually decreases the ability of osteoblasts to mineralize and increases the bone-resorptive activity of osteoclasts. Adiponectin also stimulates *MMP-9* and *tartrate-resistant acid phosphate (TRAP)* expression and increases IL-8 secretion in osteoblasts. However, in RA-induced human bone tissue, adiponectin inhibited osterix expression and induced *osteoprotegrin* mRNA expression, which hinders bone formation [105]. In summary, all these studies suggest that high adiponectin levels may have pathological effects on RA patients, as adiponectin stimulates chronic inflammation of RA.

#### 5.2.2. Pro-Inflammatory Role of Adiponectin in Chronic Kidney Disease (CKD)

Various studies indicate that CKD patients have an elevated adiponectin level, particularly those with end stage renal disease. Studies have reported that the total adiponectin level in patients with end stage renal disease is three-fold higher than that of the normal group [106,107]. Additionally, a high level of adiponectin in CKD patients has a positive relationship with cardiovascular mortality, which is contradictory to adiponectin’s anti-atherogenic and cardioprotective role [108,109]. Furthermore, CKD patients have systemic low-grade chronic inflammation that seems to play a key role in triggering renal injury and promoting CKD [110,111].

The reason why a high serum level of adiponectin has negative effects on CKD patients is controversial. CKD patients show elevated levels of the C-reactive protein (CRP), IL-6, and TNF-α [112] and have aberrant TLR-4 activation. In experiments conducted on 29 CKD patients in stage 5 and 14 healthy controls, CKD patients had a high level of TLR-4 activation compared to healthy controls. Consistent with increased in vivo TLR-4 activation in CKD patients, in vitro TLR-4 stimulation induced TNF-α and NF-κB activation in C2C12 cells. This indirectly suggests that muscle inflammation of CKD patients could be promoted by TLR-4 activation [113]. Adiponectin represses the signaling pathway of TLR-4 activation by stimulating mir-711 signaling pathway, which then leads to anti-inflammatory effects [35]. However, the expectation that CKD patients have an anti-inflammatory state is not consistent with the actual high inflammation of CKD patients. For example, CKD patients are expected to have an anti-inflammatory response due to the high serum level of adiponectin, but they actually show elevated TLR-4 activation and high inflammation. This discrepancy could be explained in three ways. First, CKD’s severe inflammation overwhelms the anti-inflammatory effects of adiponectin. Second, some factors block adiponectin signal transduction [114]. Third, adiponectin may have pro-inflammatory effects in CKD patients. Furthermore, several recent studies have pointed out that adiponectin can be pro-inflammatory or anti-inflammatory according to the isoform ratio because the isoforms have different physiological functions [115]. The reason why the adiponectin serum level is increased in CKD patients is still controversial. The differential effects of these isoforms need to be investigated further.

#### 5.2.3. Pro-Inflammatory Role of Adiponectin in Inflammatory Bowel Disease

Inflammatory bowel disease (IBD) is comprised of two chronic inflammatory diseases known as Crohn’s disease and ulcerative colitis. The incidence of IBD is increasing worldwide. Though its etiology is largely unknown, IBD is thought to result from specific genetic, environmental, or microbial factors and immune responses [116]. Patients with IBD have abnormal circulating levels of adipokines. IBD patients have higher levels of adiponectin and resistin and a lower level of leptin based on a study with 100 patients and 60 controls [117]. Adiponectin increased proinflammatory cytokines like IL-6 and MIP-2 production in colon tissue. Several studies demonstrated that adiponectin-AdipoR signaling is involved in colonic inflammation. First, adiponectin increased pro-inflammatory cytokine production in the colon tissue. Furthermore, *adiponectin*-knockout mice were protected from dextran sulfate sodium (DSS)-induced colitis, while wild type mice had severe colitis. Treating *adiponectin*-knockout mice with adiponectin induced inflammation [118]. Additionally, another study showed that treating *AdipoR1* transgenic mice with adiponectin induced colitis through increased expression of *COX-2* and chemokines such as *CXCL1, CXCL2*, and *CXCL5* in neutrophils. This suggests that adiponectin-AdipoR1 signaling could recruit neutrophils into colonic tissue and induce inflammation [119]. Opposite outcomes were reported in another *adiponectin*-knockout mouse species. DDS induced more severe colitis in adiponectin-knockout mice than wild mice by increasing the number of B cells and the expression of inflammatory cytokines like *IL-4* and *IL-6* [120]. Consistent with the result that adiponectin induced colonic inflammation in mice models, patients with IBD usually have higher adiponectin and IL-6 levels, which activate the signal transducer and activator of transcription 3 (STAT3). Importantly, STAT3 activation induces apoptosis resistance of lamina propria T lymphocytes, which is considered a main factor of chronic inflammation in IBD [121]. All these studies indirectly demonstrate that adiponectin plays important roles in inflammation of colonic tissue through stimulation of AdipoR signaling.

Adiponectin is increased in some diseases with chronic inflammation such as RA, CKD, IBD, type 1 diabetes, cystic fibrosis, and systemic lupus erythematosus (SLE) [76]. In addition, other studies showed that patients with systemic autoimmune diseases have elevated adiponectin serum levels [122,123]. However, the reasons why adiponectin is positively associated with autoimmune diseases have not been elucidated, and adiponectin’s role in inflammation remains controversial.

## 6. Differential Effects of Adiponectin Isoforms and Their Controversies

### 6.1. Issues Related to HMW: Pro-Inflammatory or Anti-Inflammatory

Studies revealed that both LMW and HMW induce cell apoptosis in THP-1 cells by activating *AMPK* and suppressing *macrophage scavenger receptor (MSR)* mRNA expression. This means that adiponectin isoforms have common effects on human monocytic cells like cell apoptosis and activation of AMPK. Additionally, HMW induced activation of IL-6 in human monocytic cells, but it could not suppress LPS-induced IL-6 secretion in LPS-treated macrophages. In contrast, LMW did suppress IL-6 secretion and stimulate IL-10 secretion in LPS-treated macrophages. LMW also blocked nuclear translocation of NF-κB p65. These results mean that only LMW has anti-inflammatory effects, while HMW seems to activate pro-inflammatory factors [124,125]. Other studies support the pro-inflammatory effects of HMW [115]. When HMW was added to peripheral blood mononuclear cells (PBMCs) and microvascular endothelial cells (MECs), secretion of MCP-1 and IL-8 increased, but this was not observed in human glomerular mesangial cells (GMCs). However, LMW did not show a pro-inflammatory effect on the above cells, which means that only HMW has pro-inflammatory effects on specific cells [115]. Furthermore, when the adiponectin gene was mutated to form LMW but not HMW, NF-κB activation was inhibited. This suggests that only HMW triggers activation of NF-κB [62]. In contrast to the above results, some studies indicate that HMW has both pro-inflammatory and anti-inflammatory effects. HMW activated AMPK and phosphorylated eNOS, which results in an anti-inflammatory effect. However, HMW increased TNF-α-induced NF-κB activation, which induces pro-inflammatory action. Taken together, these findings suggest that adiponectin-induced AMPK/eNOS activation and repressed NF-κB activation [126]. In conclusion, the above results show that adiponectin isoforms have differential physiological effects on inflammation.

In the case of RA patients, the physiological role of HMW is well established. In particular, HMW adiponectin up-regulated release of IL-6, IL-8, and MMP-3 in RA synovial fibroblasts (FLS). However, LMW did not stimulate this secretion. This means that HMW both triggers inflammation and stimulates the release of IL-6 and IL-8, which is associated with pro-destructive effects in RA FLS [127]. The secretion of TGFβ, IL-6, IL-1Ra, PGE_2_, IL-8, and VEGF was considerably increased in HMW-treated RA-adipose mesenchymal stem cells but not in LMW-treated cells [128]. Furthermore, the ratio of three full-length adiponectin isoforms was compared in the serum of RA patients. The stimulated pro-inflammatory factors were investigated in RA FLS, which were treated with the three isoforms. This study showed that HMW/MMW induced secretion of the strongest inflammatory factors of TNF-α, VEGF, and PGE_2_, which deteriorate RA [129]. Taken together, the above studies demonstrate that HMW has pro-inflammatory effects at least in cases of specific diseases like RA.

However, several studies suggest that HMW is anti-inflammatory. In particular, the HMW ratio in serum increased significantly when sepsis patients showed clinical improvement. In contrast, the HMW ratio was inhibited in the progressive stage of sepsis [130]. This suggests that HMW may mitigate inflammation. In addition, TNF-α, thiobarbiturate reactive substances, and hs-CRP decreased as the levels of HMW and total adiponectin increased in another study of sepsis [131]. This suggested that HMW decreases inflammation and oxidative stress. HMW treatment improved glucose homeostasis and reduced inflammation by regulating APPL1-AMPK-GLUT4 signaling in 3T3-L1 adipocytes subjected to glucolipotoxicity [132]. In addition, some studies indicated that HMW has a negative relationship with inflammatory markers in type 2 diabetic patients [133,134,135] and obese people [136].

**Figure 5:**
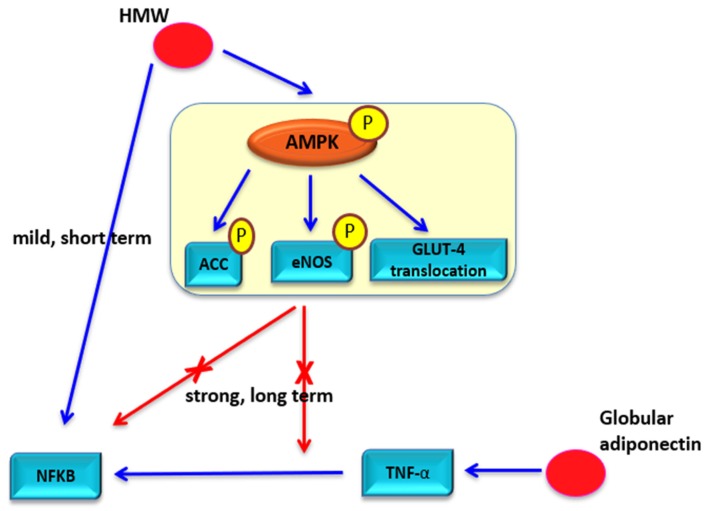
Proposed model for pro-inflammatory and anti-inflammatory signaling of HMW.

These results indicate that HMW has both pro-inflammatory and anti-inflammatory effects by activating two different signaling pathways known as AMPK and NF-κB. As shown in Figure 5, HMW induces an anti-inflammatory reaction through the AMPK-GLUT4 and AMPK-eNOS signaling pathway and a pro-inflammatory reaction through the NF-κB signaling pathway. Under normal conditions, HMW seems to have anti-inflammatory effects because multiple AMPK signaling pathways counteract NF-κB signaling pathways stimulated by HMW. Specifically, HMW stimulates NF-κB migration into the nucleus in a dose-dependent manner for a short time. Then, the increased NF-κB level stimulated by HMW is dose-dependently down-regulated by AMPK signaling pathways for longer times [126]. Thus, the decrease in adiponectin serum level leads to chronic inflammation in patients with metabolic diseases. The reason for this is that scarcity of HMW does not induce stimulation of AMPK signaling and leads to NF-κB signaling. This results in increased oxidative stress and lipid accumulation, which are the main causes of inflammation.

In contrast to metabolic disorders, a high HMW serum level in autoimmune diseases seems to exacerbate chronic inflammation through a pro-inflammatory reaction of HMW. Patients with autoimmune disease show continuous production of HMW, and the severity of their symptoms had a positive relationship with the serum HMW level [122,123]. In addition, both NF-κB and TNF-α are consistently stimulated in patients with autoimmune diseases [137]. A plausible mechanism is provided in Figure 5. First, high serum HMW level leads to strong activation of NF-κB signaling pathways because HMW stimulates NF-κB in a dose-dependent manner. The instant inflammatory reaction caused by short-term elevation of HMW is counteracted as the HMW level returns to its normal state. However, persistent production and activation of HMW in autoimmune patients maintain NF-κB signaling. Second, autoimmune disease patients have high serum levels of both HMW and globular adiponectin. Globular adiponectin seems to stimulate TNF-α-NF-κB signaling [138]. Since HMW and globular adiponectin seem to have a synergistic effect and result in elevated activation of NF-κB signaling. Third, HMW exerts anti-inflammatory effects as AMPK signaling enhances lipid metabolism and reduces inflammation. Disruption of lipid metabolism and accumulation of the lipid in tissues could be the main causes of inflammation in metabolic disease. In contrast, disrupted lipid metabolism does not seem to be directly related to autoimmune diseases but is induced by immune cells but not by lipid metabolism. This indirectly suggests that enhanced AMPK signaling would not counteract stimulation of TNF-α-NF-κB signaling in autoimmune diseases.

HMW seems to exert both pro-inflammatory and anti-inflammatory effects by activating two signaling pathways. HMW-mediated AMPK-ACC, AMPK-eNOS, and AMPK-GLUT4 signaling pathways enhance lipid metabolism, which decreases oxidative stress and TNF-α activation. In normal conditions, anti-inflammatory reactions stimulated by AMPK signaling are stronger and more persistent than pro-inflammatory effects by NF-κB. Thus, HMW exerts an overall anti-inflammatory effect by the overwhelming pro-inflammatory effect.

### 6.2. Globular Adiponectin with a Pro-Inflammatory Effect

Many studies have indicated that globular adiponectin may have pro-inflammatory effects. *IL-8, GM-CSF*, and *MCP-1* in mRNA levels significantly increased when globular adiponectin was added to colonic epithelial cells. Globular adiponectin stimulated nuclear translocation of NF-κB through phosphorylation of ERK and p38 MAPK signaling pathways [139]. In particular, NF-κB was very strongly activated by globular adiponectin, which is stronger than activation by TNF-α and LPS [62]. It also enhanced fMLF-induced ROS production in human phagocytes and showed synergistic effects with TNF-α-mediated *NOX-2* expression [99]. However, globular adiponectin may have anti-inflammatory effects by inducing AMPK signaling pathways. Globular adiponectin suppressed LPS-primed inflammasome activation by stimulating AMPK signaling pathways [140]. Additionally, rat chondrocyte was protected from apoptosis by globular adiponectin-induced AMPK-mTOR signaling [141].

## 7. Conclusions

Previous studies have shown the insulin-sensitizing, anti-atherogenic, and anti-inflammatory effects of adiponectin. Furthermore, various effects on different tissues were shown to be based on tissue-specific signaling pathways of adiponectin. However, severity of inflammation in autoimmune diseases was positively associated with a level of adiponectin, which suggests that adiponectin could play a role in the pro-inflammatory response. In particular, patients with metabolic diseases like T2D and obesity showed chronic inflammation with a low level of serum adiponectin, while autoimmune disease patients (including those with RA and CKD) had chronic inflammation with a high level of serum adiponectin. The reasons why RA, CKD, IBD, and SLE patients have a high serum adiponectin level during chronic inflammation have not been shown. This review suggests the multifaceted role of adiponectin and the different signaling roles of its isoforms as a plausible explanation. HMW seems to activate both pro-inflammatory and anti-inflammatory signaling pathways, while globular adiponectin is known to stimulate pro-inflammatory effects. Since adiponectin is a key hormone for various physiological reactions, overall inhibition of the adiponectin reaction would bring side effects. Thus, specific isoforms would be ideal targets for controlling inflammation of metabolic and autoimmune diseases. Further research on the physiological role of each isoform and their complicated interactions with signaling molecules needs to be conducted to target the specific isoforms for therapeutics.

## Abbreviations

WAT	white adipose tissue
HMW	high-molecular weight adiponectin
MMW	middle-molecular weight adiponectin
LMW	low-molecular weight adiponectin
AMPK	adenosine monophosphate-activated protein kinase
P38 MAPK	p38 mitogen-activated protein kinase
PPARα	peroxisome proliferator-activated receptor-α
APPL	adaptor protein containing a pleckstrin homology domain
NF-κB	nuclear factor κ-light-chain-enhancer of activated B cells
TNF-α	tumor necrosis factor-α
NO	nitric oxide
AdipoR	adiponectin receptor
T2B	type 2 diabetes
RA	rheumatoid arthritis
CKD	chronic kidney disease
IRS	insulin receptor substrate
IR	insulin receptor tyrosine kinase
TLR	toll-like receptor
